# Efficacy of the bumped kinase inhibitor BKI-1708 against the cyst-forming apicomplexan parasites *Toxoplasma gondii* and *Neospora caninum in vitro* and in experimentally infected mice

**DOI:** 10.1016/j.ijpddr.2024.100553

**Published:** 2024-06-19

**Authors:** Maria Cristina Ferreira de Sousa, Dennis Imhof, Kai Pascal Alexander Hänggeli, Ryan Choi, Matthew A. Hulverson, Samuel L.M. Arnold, Wesley C. Van Voorhis, Erkang Fan, Sánchez-Sánchez Roberto, Luis M. Ortega-Mora, Andrew Hemphill

**Affiliations:** a Institute of Parasitology, Vetsuisse Faculty, University of Bern, Switzerland; b Graduate School for Cellular and Biomedical Sciences (GCB), University of Bern, Switzerland; c Center for Emerging and Re-emerging Infectious Diseases (CERID), Division of Allergy and Infectious Diseases, Department of Medicine, University of Washington, Seattle, WA, USA; d Department of Pharmaceutics, University of Washington, Seattle, WA, USA; e Department of Biochemistry, University of Washington, Seattle, WA, USA; f SALUVET, Animal Health Department, Faculty of Veterinary Sciences, Complutense University of Madrid, Ciudad Universitaria s/n, Madrid, Spain

**Keywords:** *Neospora*, *Toxoplasma*, Drug treatment, *In vitro* culture, Electron microscopy, *In vivo* efficacy, Mouse model, PCR

## Abstract

*Toxoplasma gondii* and *Neospora caninum* are major worldwide morbidity-causing pathogens. Bumped kinase inhibitors (BKIs) are a compound class that has been optimized to target the apicomplexan calcium-dependent protein kinase 1 (CDPK1) – and several members of this class have proven to be safe and highly active *in vitro* and *in vivo*. BKI-1708 is based on a 5-aminopyrazole-4-carboxamide scaffold, and exhibited *in vitro* IC_50_ values of 120 nM for *T. gondii* and 480 nM for *N. caninum* β-galactosidase expressing strains, and did not affect human foreskin fibroblast (HFF) viability at concentrations up to 25 μM. Electron microscopy established that exposure of tachyzoite-infected fibroblasts to 2.5 μM BKI-1708 *in vitro* induced the formation of multinucleated schizont-like complexes (MNCs), characterized by continued nuclear division and harboring newly formed intracellular zoites that lack the outer plasma membrane. These zoites were unable to finalize cytokinesis to form infective tachyzoites. BKI-1708 did not affect zebrafish (*Danio rerio*) embryo development during the first 96 h following egg hatching at concentrations up to 2 μM. Treatments of mice with BKI-1708 at 20 mg/kg/day during five consecutive days resulted in drug plasma levels ranging from 0.14 to 4.95 μM. *In vivo* efficacy of BKI-1708 was evaluated by oral application of 20 mg/kg/day from day 9–13 of pregnancy in mice experimentally infected with *N. caninum* (NcSpain-7) tachyzoites or *T. gondii* (TgShSp1) oocysts. This resulted in significantly decreased cerebral parasite loads and reduced vertical transmission in both models without drug-induced pregnancy interference.

## Highlights

• BKI-1708 is a novel CDPK1 inhibitor with profound activity against *Neospora* and *Toxoplasma*.
• *In vitro*, BKI-1708 induces the formation of multinucleated complexes.
• BKI-1708 treatment does not interfere in zebrafish embryo development.
• BKI-1708 efficiently inhibits vertical transmission of both parasites and increases pup survival.

## Graphical abstract

**Figure:**
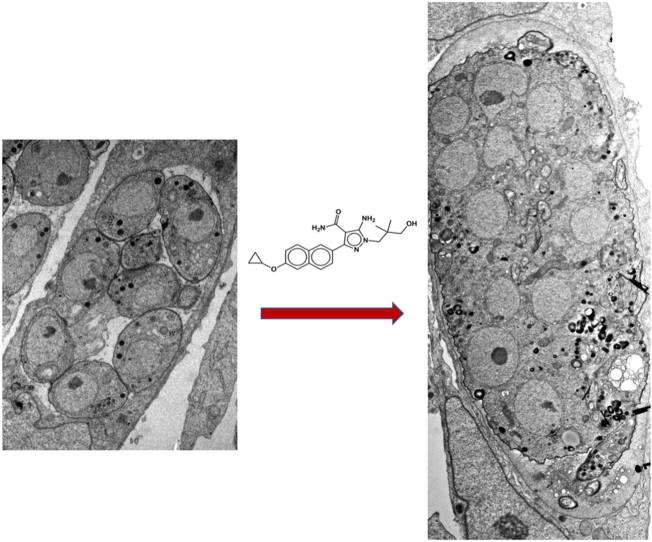

## 1 Introduction

*Toxoplasma gondii* and *Neospora caninum* are two closely related pathogens belonging to the subphylum Apicomplexa. Both are known to inflict problems during pregnancy and/or upon immunosuppression. *T. gondii* is amongst the most successful parasites worldwide. Toxoplasmosis is one of the most common global zoonotic diseases with an enormous impact on human and veterinary health. Its veterinary importance is linked to reproductive failure and abortion, especially in small ruminants. In humans, toxoplasmosis symptoms are mild and subside within a few days to weeks in most cases. However, in immunocompromised patients, and upon primary infection during pregnancy, toxoplasmosis can become life threatening (Dubey, 2021). *N. caninum* is one of the main causes of abortion in cattle, leading to important economic losses in beef and dairy industries, and is also relevant for dogs, causing life-threatening neuromuscular disease (Schares, 2017).

Both parasites share a similar life cycle that comprises three infective stages: sporozoites, which are the end product of a sexual development taking place in the definitive hosts (canids for *N. caninum*, felines for *T. gondii*), rapidly proliferating tachyzoites that inflict acute disease, and slowly dividing bradyzoites that form tissue cysts. The definitive hosts harbor sexual stages in the intestine, resulting in the production of oocysts that are shed in feces. Following sporulation in the environment and oral uptake, sporozoites can cause infection in both definitive and intermediate hosts (environmental pathway). Alternatively, carnivorous intermediate hosts can get infected through ingestion of bradyzoites in tissue cysts, or ruminants can get infected by ingesting placental tissue after delivery (meat-borne pathway) (Schares, 2017; Dubey, 2021). Bradyzoites are formed during chronic infection as a response to physiological and immunological stress exerted by immunocompetent hosts, resulting in the formation of tissue cysts.

Under normal circumstances, *T. gondii* infection in humans and animals will proceed largely unnoticed, with two exceptions. First, primary infection during pregnancy can lead to transplacental infection of the fetus, which can cause malformations or even abortion. Secondly, in hosts persistently infected with *T. gondii*, immunosuppression often leads to rapid re-differentiation of bradyzoites into tachyzoites and dissemination of parasites into multiple organs including the brain, causing life-threatening pathologies. *N. caninum* infection has not been reported in humans, but in multiple animal species (Dubey et al., 2017), with cattle being most economically relevant. Infection of cattle occurs by two routes: post-natal ingestion of sporozoite-containing oocysts, which excyst and enter intestinal epithelium and cells of the reticuloendothelial system, undergo initial dissemination and proliferation, and form bradyzoites and tissue cysts in brain and muscle, leading to subclinical and persistent infection; and vertical/transplacental transmission from dam to fetus, which can occur via endogenous or exogenous transplacental transmission (EnTT and ExTT, respectively). EnTT occurs in chronically infected dams after recrudescence of infection during pregnancy, while ExTT takes place after primary infection of pregnant dams via ingestion of oocysts. In both cases, tachyzoites cross the placenta and infect the fetus, leading to either abortion or birth of weak calves, or asymptomatic but persistently infected calves that can then transmit the disease in successive gestations. In some cases, no transmission takes place. EnTT in cattle is highly efficient and can occur repeatedly in successive pregnancies. With time, a large portion of a herd can get infected with this parasite, with persistently infected dams at much higher risk to abort compared to non-infected dams, leading to endemic abortions associated with high economical losses. ExTT occurs less frequently and leads to an epidemic abortion pattern (Dubey et al., 2017).

To this day, there is no safe and effective drug against bovine neosporosis on the market. Furthermore, drugs used against toxoplasmosis are limited, often exhibiting adverse side effects or poor efficacy (Doggett et al., 2014; Hemphill et al., 2016). Therefore, novel therapeutic options are being investigated, amongst which stands a promising class of anti-protozoan drugs with proven efficacy *in vivo* and *in vitro* against several apicomplexans: bumped kinase inhibitors (BKIs) (Choi et al., 2020).

BKIs have been optimized to selectively target calcium-dependent protein kinase 1 (CDPK1), important for motility and invasion and exclusive to apicomplexan parasites (Huang et al., 2019). Calcium-mediated signaling plays a major role in apicomplexans since these parasites rely on calcium as a second messenger to regulate a variety of essential cellular processes (Lourido et al., 2013) such as cytokinesis, intracellular proliferation, stage differentiation (Wei et al., 2013) and specific events that ensure life cycle progression such as secretion, gliding motility, and host cells invasion (Jacot and Soldati-Favre, 2012). Apicomplexan CDPK1 has become a promising candidate for therapeutic intervention, being conserved among apicomplexans but absent from mammalian hosts (Lourido et al., 2013). CDPKs with small gatekeeper residues in the ATP binding-site are inhibited by BKIs (Keyloun et al., 2014). Almost all mammalian kinases have bulkier gatekeepers, yet a naturally occurring small glycine gatekeeper residue in the ATP-binding site of apicomplexan CDPK1 opens up a hydrophobic pocket that enables BKIs to bind, rendering this class of compounds highly selective and active against apicomplexans (Huang et al., 2019; Van Voorhis et al., 2017). However, more recent studies suggested that some BKIs could also affect other targets. Affinity chromatography using matrix-bound BKIs on parasite and host cell extracts combined with MS and proteomics identified many BKI-binding proteins involved in RNA-binding and modification, such as ribosomal proteins and proteins in RNA splicing, suggesting that essential pathways such as translation and RNA processing could also be affected (Müller et al., 2022; Ajiboye et al., 2024).

Previous studies revealed that some, but not all, BKIs were able to penetrate the blood-brain barrier, the placenta, and the eyes, as required for an ideal drug against a systemic infections such as toxoplasmosis and neosporosis (Van Voorhis et al., 2017). BKIs have proven to be safe and highly active *in vitro* and *in vivo* against several apicomplexans (Choi et al., 2020), including *T. gondii* and *N. caninum*. Reports on the efficacy of BKI-1294 (Winzer et al., 2015), BKI-1517 and -1553 (Müller et al., 2017b; Sánchez-Sánchez et al., 2018a) and BKI-1748 (Imhof et al., 2021; Sánchez-Sánchez et al., 2024) against these closely related apicomplexans opened the door for further testing of these novel kinase inhibitors. BKIs structurally based on an 5-aminopyrazole-4-carboxamide (AC) scaffold, such as BKI-1708 investigated in this study, are considered to be safer than pyrazolopyrimidine (PP) based BKIs, exhibiting low interference with the human ether-á-go-go related gene (hERG) and reduced cardiovascular liability (Huang et al., 2019; Choi et al., 2020).

The following study describes the *in vitro* activity of BKI-1708 against *T. gondii* and *N. caninum* tachyzoites, and the *in vivo* pharmacokinetic profile and efficacy in respective pregnant and non-pregnant mouse models.

## 2 Materials and methods

### 2.1 Cell culture media, biochemicals, BKI-1708

If not stated otherwise, all tissue culture media were purchased from Gibco-BRL (Zürich, Switzerland) and biochemicals from Sigma (St. Louis, MO, USA). BKI-1708 was synthesized in the Department of Biochemistry of the University of Washington, USA (Huang et al., 2019) and scaled up by WuXi Apptec Inc., Wuhan, China to >98% purity by LC/MS-MS and NMR, being provided as powder stored at room temperature. For *in vitro* studies, stock solutions of 20 mM were prepared in dimethyl-sulfoxide (DMSO) and stored at −20 °C. For *in vivo* experiments, the compound was emulsified in corn oil, followed by administration to mice by oral gavage.

### 2.2 Host cells and parasites

Human foreskin fibroblasts (HFF; ATCC, PCS-201-010™) and BALB/c dermal fibroblasts (CELLNTEC AG, Bern, Switzerland) were maintained in Dulbecco's modified Eagle medium (DMEM) supplemented with 10% heat-inactivated and sterile filtered fetal calf serum (FCS), 50 U of penicillin/ml, and 50 μg streptomycin/ml as previously described (Winzer et al., 2020a). *N. caninum* strain NcSpain-7 and the transgenic strain *N. caninum* Nc1 expressing β-galactosidase (Nc-β-gal) were grown and maintained in HFF (Aguado-Martínez et al., 2016). *T. gondii* ME49 and *T. gondii* β-gal tachyzoites (RH background, Tg-β-gal) were cultured as previously described (Winzer et al., 2015). *T. gondii* oocysts of the type II isolate *T. gondii* sheep Spain 1 (TgShSp1) were generated at the Complutense University of Madrid, Spain, and were stored at 4 °C (Sánchez-Sánchez et al., 2019).

### *2.3* *Cytotoxicity and anti-*T. gondii*/anti-*N. caninum *efficacy assessments in vitro*

Transgenic Tg-β-gal and Nc-β-gal were used to assess the *in vitro* efficacy of BKI-1708 as described previously (Anghel et al., 2020). In short, HFF monolayer cultures grown in 96-well plates at 37 °C/5%CO_2_ were infected and treated either concomitantly (pre-infection) or 3 h after infection (post-infection) with different BKI-1708 concentrations ranging from 0.0078 μM to 1 μM. Cultures were further maintained at 37 °C/5% CO_2_ and the IC_50_ value was determined at 72 h post-infection. The half-maximal drug concentration that inhibits tachyzoite proliferation by 50% (IC_50_), was calculated by regression analysis of the logit-log transformation of relative growth using the corresponding software tool contained in the Excel software package (Microsoft, Seattle, WA) as described previously (Müller and Hemphill, 2013). The effects of the compound on HFF viability were assessed by resazurin reduction assay, following the protocols of previous studies (Anghel et al., 2020).

### 2.4 Transmission electron microscopy (TEM)

HFF were grown to confluence in T25 flasks in culture medium at 37 °C/5% CO_2_, and were infected with 1 × 10^7^ *T. gondii* ME49 tachyzoites. At 24h post-infection, the medium was supplemented with BKI-1708 at 2.5 μM. The medium was removed after 24, 48, 72 and 96 h of continuous treatment at 37 °C/5%CO_2_, cultures were once washed with 100 mM sodium cacodylate, pH 7.3. Fixation was carried out in 100 mM cacodylate buffer containing 2% glutaraldehyde for 10 min, and adherent cells were carefully removed with a cell scraper. Following 2–4 h of fixation at room temperature, samples were centrifuged and post-fixed in 2% cacodylate buffer containing 2% osmium tetroxide during 2 h. Following several washes in distilled water, specimens were dehydrated through a graded series of ethanol (30, 50, 70, 90 and 3 × 100%), and embedded in Epon 812 epoxy resin as previously described (Schlange et al., 2023). Polymerization of the resin was carried out at 60 °C overnight. Sections of 80 nm thickness were cut on an ultramicrotome (Reichert and Jung, Vienna, Austria) and were transferred onto formvar-carbon-coated 200 mesh nickel grids (Plano GmbH, Marburg, Germany), and were stained with Uranyless® and lead citrate (Electron Microscopy Sciences, Hatfield PA, USA). Imaging of the specimens was performed on a FEI Morgagni TEM equipped with a Morada digital camera system (12 Megapixel) operating at 80 kV.

### 2.5 Zebrafish embryotoxicity assay

The zebrafish embryotoxicity assay was used to assess the potential embryotoxicity of BKI-1708 during the early developmental stages by exposing embryos to different drug concentrations (from 0.2 to 50 μM) for 96 h and monitoring of malformations or early embryonic death every 24h by light microscopy. Evaluation of malformations was done in a blinded fashion (e.g. the evaluator did not know what samples he was inspecting). An impact score based on the observations was calculated in relation to zebrafish embryos developing in water and embryos developing in the compound solvent (DMSO) as described in Anghel et al. (2020).

### 2.6 Ethical statement

Protocols involving animals were approved by the Animal Welfare Committee of the Canton of Bern under licenses BE117/2020 and BE048/2024. Animals were handled in strict accordance with practices to minimize suffering. BALB/c and CD1 mice, 6 weeks of age, were purchased from Charles River (Sulzberg, Germany), and were maintained in a common room under controlled temperature and a 14 h/10 h light/dark cycle in cages of not more than 4 animals each. Mice were housed in the facility for two weeks for adaptation prior to the experiments and procedures were carried out according to the guidelines of the animal welfare legislation of the Swiss Veterinary Office.

### 2.7 Pharmacokinetics (PK) of BKI-1708 in BALB/c mice

18 non-pregnant BALB/c mice and 7 pregnant BALB/c mice of 8 weeks of age were used. In non-pregnant mice BKI-1708 was dosed at 20 mg/kg in 0.1 mL of sterile corn oil and administrated to mice by oral gavage, followed by blood sampling (tail vein) at several time points: 30min, 1, 2, 5, 7 and 24 h after the treatment. Pregnant mice were treated with the compound for 5 days and sampled only once on the last day of treatment. Whole blood was collected in ethylenediaminetetraacetic acid (EDTA) tubes and incubated at room temperature for 30 min and centrifuged in an Eppendorf tube at 1200 rpm, 4 °C for 10 min to obtain plasma. Samples were stored at −20 °C and the BKI-1708 plasma concentrations were measured. For this, the compound was extracted from the plasma samples using acetonitrile/0.1% formic acid containing an internal standard. A standard mix in control plasma was prepared for comparison and quantification. BKI-1708 was quantified by liquid chromatography-mass spectrometry/mass spectrometry analysis as previously described by Huang et al. (2019).

### 2.8 Assessment of BKI-1708 efficacy in CD1 mice orally infected with TgShSp1 oocysts

Efficacy assessment of BKI-1708 in orally TgShSp1 oocyst-infected CD1 mice was done as previously described for BKI-1748 (Imhof et al., 2021). A total of 63 CD1 mice were used: 42 females and 21 males, all 8 weeks of age. Females were estrus synchronized by exposure to male pheromones, by placing embedding from male cages into their cages for 3 consecutive days, taking advance of the Whitten effect. Subsequently, one male was housed with 2 females during 96 h for mating. Following mating, females were distributed randomly into 4 experimental groups: i) negative control group (C-) with non-infected (challenged with PBS) and non-treated mice (corn oil was used as placebo) (n = 6), ii) positive control group (C+) with infected but non-treated mice (corn oil used as placebo) (n = 12) and two treatment groups: iii) BKI-1708 dosed at 5 mg/kg/day (n = 12) and iv) BKI-1708 dosed at 20 mg/kg/day (n = 12). Mice were orally infected with 100 TgShSp1 oocysts suspended in PBS on day 7 post-mating and daily treatments were started 2 days post-infection for 5 consecutive days. For this, oocysts were stored at 4 °C and were counted and diluted on the day of challenge. The compound was dissolved in sterile corn oil and heated up to 37 °C prior to treatment by oral gavage. Mice were separated according to weight 18 days post mating: pregnant mice were placed in single cages to give birth and raise their pups, while non-pregnant mice were housed in cages in groups of 3–4. All pups were born on days 20–24 post-mating. The mice were closely monitored for health-related events during pregnancy and during post-partum (p.p). Data on litter sizes as well as neonatal and postnatal mortality rates were noted. Mice and surviving pups were sacrificed 30 days p.p. in a chamber by isoflurane/CO₂. Blood of adults was collected via cardiac puncture and serum was extracted for confirmation of infection by IgG measurement by enzyme-linked immunosorbent assay (ELISA). Brain and eyes were aseptically removed and stored at −20 °C to determine parasite burden by RT-qPCR (Sánchez-Sánchez et al., 2019; Imhof et al., 2021).

### 2.9 BKI-1708 efficacy in BALB/c mice infected with NcSpain-7 tachyzoites

A total of 57 BALB/c mice were used: 38 females and 19 males, all 8 weeks old. Mating was performed as described above and females were randomly distributed into 3 experimental groups: i) negative control group (C-) with non-infected (challenged with BALB/c dermal fibroblasts suspended in PBS) and non-treated mice (corn oil was used as placebo) (n = 10), ii) positive control group (C+) with infected but non-treated mice (PBS used as placebo) (n = 14): iii) treatment group: infected mice treated with BKI-1708 dosed at 20 mg/kg/day (n = 14). For the infection, NcSpain-7 tachyzoites were cultured in HFF at 37 °C/5% CO₂ and transferred to BALB/c fibroblasts to be grown under the same conditions for three days prior to infection. Parasites were then collected and counted, and each mouse was infected by subcutaneous injection with 10⁵ NcSpain-7 tachyzoites suspended in 100 μL PBS (Arranz-Solís et al., 2015). Infection took place on day 7 post-mating. Pregnant and non-pregnant mice were separated as described for the toxoplasmosis model and evaluated for clinical signs and mortality rates. Pregnant mice gave birth on days 20–23 post-mating. On day 30 p.p., adult mice and surviving pups were sacrificed in a chamber by isoflurane/CO₂, and brain and blood were collected for assessment of the burden and IgG measurements, respectively.

### 2.10 Determination of cerebral parasite load by real-time PCR

Brains of adult mice and surviving pups were collected directly after sacrifice and parasitic load was quantified by real-time PCR specific for *T. gondii* (Costa et al., 2000) and *N. caninum* (Müller et al., 2002). Given the tropism of *T. gondii* for the eye, eyes of adult mice infected with *T. gondii* were also collected. DNA was extracted from all samples using the Nucleospin Kit (Macherey-Nagel, Oensingen, Switzerland) and QuantiFluor dsDNA System (Promega, Madison, Wi, USA) was used to determine the DNA concentration. The DNA concentration in all samples was adjusted to 5 ng/μL with sterile DNase free water (Aguado-Martínez et al., 2019). Quantitative real-time PCR was performed using the Light Cycler® System (Roche, Basel, Switzerland). Parasite load was calculated by interpolation from a standard curve of DNA samples from 1000, 100 and 10 *T. gondii* or *N. caninum* tachyzoites included in each run.

Statistical analysis of cerebral and ocular parasite load was done by comparisons between groups by the non-parametric Kruskal-Wallis test, followed by Mann-Whitney-U test. A Kaplan-Meier survival analysis was performed to compare pup mortality over time between experimental groups by plotting survival events using the Log-rank (Mantel-Cox) test. Statistical analysis was performed using Graphpad Prism version 9.5.1 (GraphPad Software, San Diego, California, USA) (Imhof et al., 2021).

### 2.11 Measurements of anti-Toxoplasma and anti-Neospora IgG by enzyme-linked immunosorbent assay (ELISA)

Confirmation of infection was carried out by verification of seroconversion in all mice. Serum IgG titers in all adult mice were measured by ELISA as previously described for *N. caninum* (Aguado-Martínez et al., 2016) and *T. gondii* infected mice (Sánchez-Sánchez et al., 2019).

## 3 Results

### 3.1 BKI-1708 inhibits the proliferation of T. gondii and N. caninum tachyzoites in vitro

**Fig. 1:**
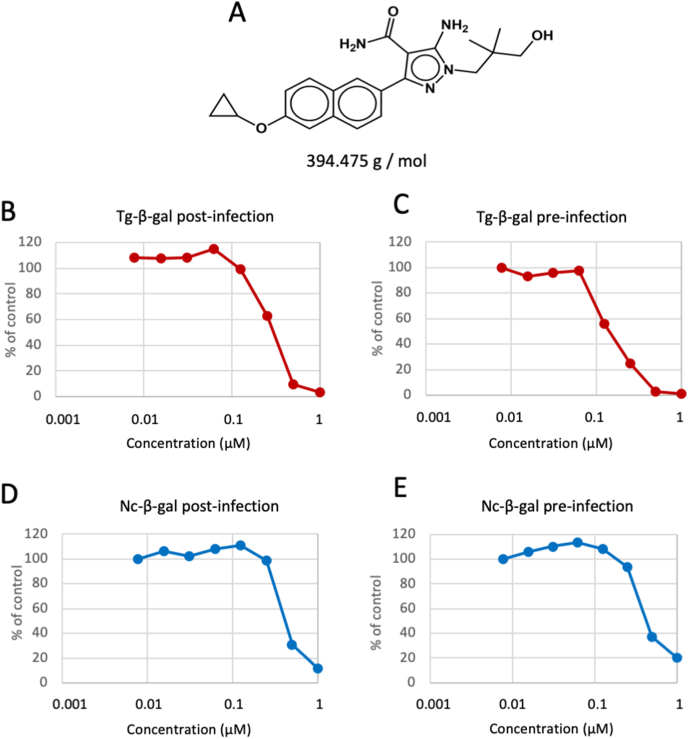
Structure and molecular weight of BKI-1708 (A), and dose-response curves for *Toxoplasma* β-gal (B, C) and *Neospora* β-gal (D, E) tachyzoites grown in HFF.

The structure of BKI-1708 is shown in Fig. 1A. The compound effectively prevented tachyzoite proliferation *in vitro*. When the compound was added concomitantly to infection of HFF monolayers with Tg-β-Gal, the IC_50_ value was 122 nM, while it was higher (327 nM) when the compound was added 3 h after the addition of Tg-β-Gal tachyzoites. For Nc-β-Gal tachyzoites, treatment concomitantly to infection resulted in an IC_50_ of 481 nM, while the IC_50_ was 964 nM when the treatment was initiated at 3 h post-infection. The viability of HFF host cells was not impaired by treatments up to 25 μM. Results are summarized in Table 1 and the respective dose-response curves are shown in Fig. 1 B-E.

**Table 1:** BKI-1708 *in vitro* activity against Tg-β-gal and Nc-β-gal. IC_50_ = half-maximal inhibitory concentration.

IC_50_ Tg-β-gal		IC_50_ Nc-β-gal		HFF viability
Pre-infection	Post-infection	Pre-infection	Post-infection	not affected
122 nM	327 nM	481 nM	964 nM	>25 μM

### 3.2 BKI-1708 treatment of T. gondii ME49 cultured in HFF transforms intracellular tachyzoites into schizont-like multinucleated complexes

**Fig. 2:**
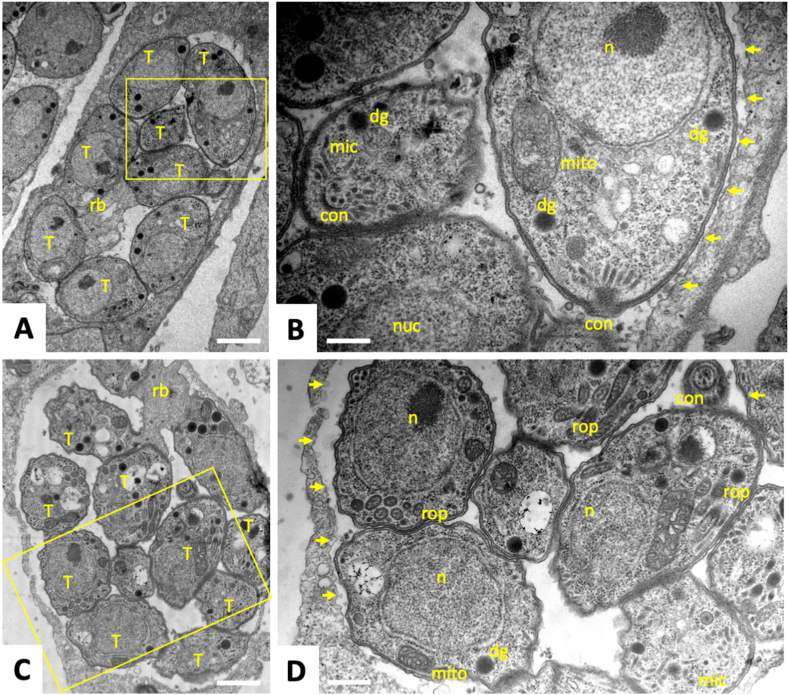
Transmission Electron Microscopy of *T. gondii* tachyzoites grown in human foreskin fibroblasts. Cells were fixed at 48 h (A, B) and 72 h (C, D) post-infection. Boxed areas in A and C are enlarged in B and D, respectively. T = tachyzoites, n = nucleus, con = conoid, mic = micronemes, rop = rhoptries, dg = dense granules, mito = mitochondrion, rb = residual body; small arrows point towards the parasitophorous vacuole membrane. Bars in (A) = 1.4 μm; (B) = 0.4 μm, (C) = 1.4 μm; (D) = 0.6 μm.

**Fig. 3:**
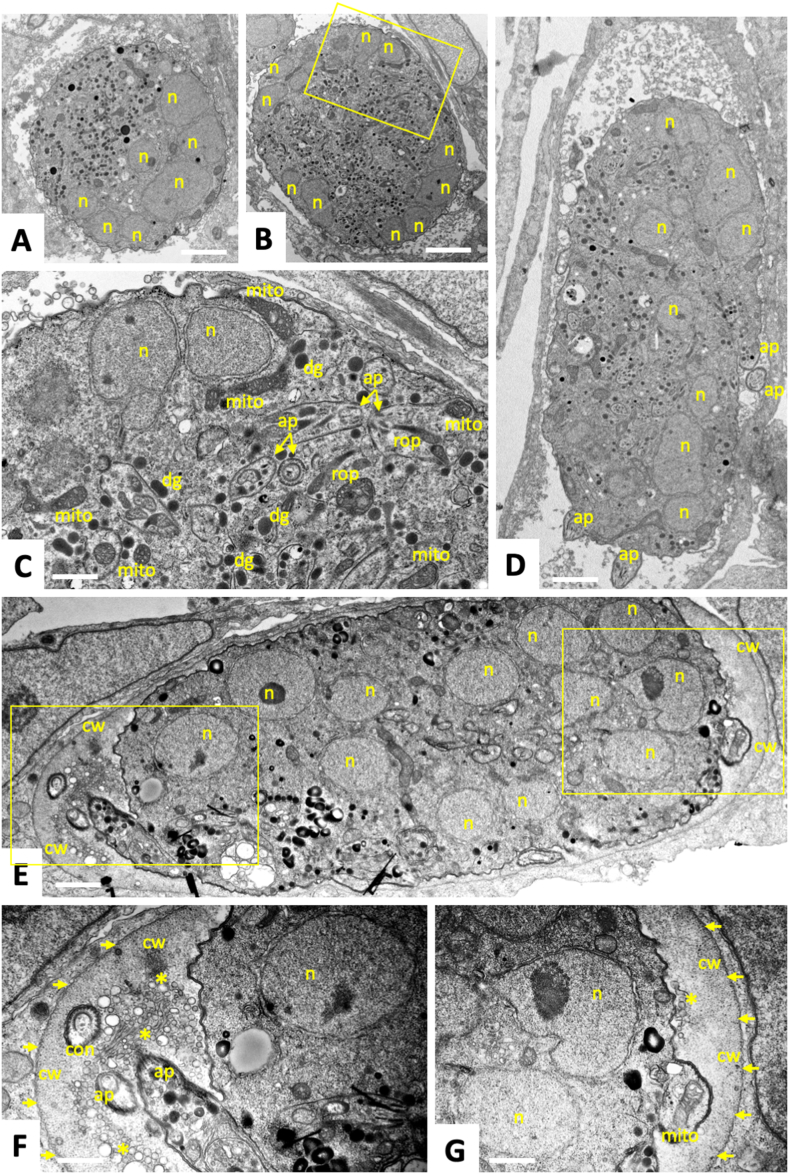
Transmission Electron Microscopy of *T. gondii* tachyzoites grown in human foreskin fibroblasts and treated with BKI-1708 (2.5 μM) during 48 h (A–C), 72 h (D) and 96 h (E–G). The boxed area in (B) is enlarged in (D), the two in (E) are enlarged in (F) and (G); n = nucleus, ap = apical part of newly formed zoite, dg = dense granules, rop = rhoptries, mito = mitochondrion, con = conoid, cw = cyst wall like structure. Small arrows point towards the parasitophorous vacuole membrane, * indicate secretory vesicles. Bars in (A) and (B) = 2.1 μm; (C) = 0.6 μm; (D) = 2.4 μm; (E) = 1.2 μm; (F) = 0.7 μm and (G) = 0.5 μm.

TEM was used to visualize *T. gondii* ME49 tachyzoites grown in HFF monolayers and to study the alterations induced by BKI-1708 treatment. In control cultures maintained in the absence of BKI-1708 for 48 (Fig. 2A and B) or 72 h (Fig. 2C, D), *T. gondii* tachyzoites proliferated intracellularly within a parasitophorous vacuole that was surrounded by a parasitophorous vacuole membrane (Fig. 2). Tachyzoites were seen either as individual parasites within the vacuole or they were still attached to a residual body (Fig. 2A–C). In any case, they were surrounded by a distinct surface membrane composed of the outer plasma membrane and the inner membrane complex. Depending on the section plane, the typical structural features of *T. gondii* tachyzoites were discernible, including the apical complex with the conoid, micronemes, dense granules and rhoptries, a single nucleus and a tubular mitochondrion with an electron dense matrix, of which usually only parts are visible on a given section. BKI-1708 treatment initiated at 4 h after infection of HFF had a pronounced effect on the overall architecture of these parasites, which became evident already after 2 days of treatment (Fig. 3A, B, C). The drug did not kill the parasites but induced the formation of intracellular multinucleated complexes (MNCs), which contained numerous nuclei that were often closely associated with each other, either to one side or to the periphery of the complex (Fig. 3A, B, C). The apparent number of these nuclei increased progressively at later time points, as did the size of these complexes (Fig. 3D–G). Besides nuclei, these complexes contained secretory organelles such as dense granules and rhoptries, mitochondria, and small apical complexes reminiscent of newly formed zoites (Fig. 3C). Mitochondria exhibited an electron dense matrix indicating that these MNCs were viable, even after longer treatment durations of 72 (Figs. 3D) and 96 h (Fig. 3E–G). At these later time points, peripheral outgrowths, which strongly resembled the apical parts of daughter zoites, were more prominently seen (Fig. 3E–G). In addition, after 96 h of treatment (Fig. 3E–G), MNCs exhibited a peripheral rim composed of electron dense material that could indicate the formation of a cyst wall-like structure. The matrix of the MNC-containing parasitophorous vacuole often contained small vesicles, most likely continuously released by the MNCs, as indicated in Fig. 3F and G. Individual tachyzoites were not detected in BKI-1708 treated cultures, especially not at the later time points.

### 3.3 Pharmacokinetics and safety assessment of BKI-1708

**Fig. 4:**
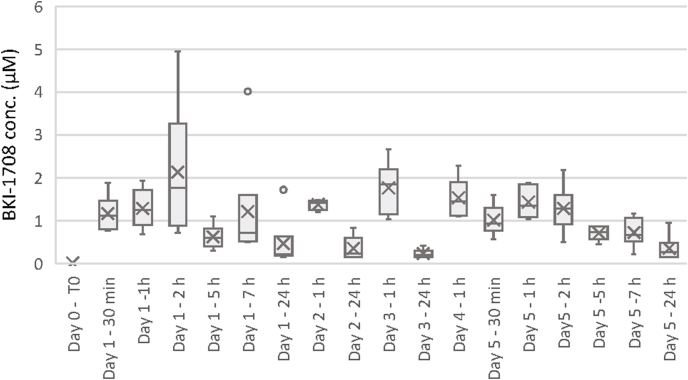
BKI-1708 concentrations in plasma of BALB/c mice treated by oral gavage with BKI-1708 suspended in corn oil at 20 mg/kg/day for 5 consecutive days. As indicated, plasma samples were obtained at several time points after treatment.

BKI-1708 concentration was measured in plasma samples from 18 non-pregnant BALB/c mice and 7 pregnant mice at different time points following treatment with 20 mg/kg/day for 5 consecutive days. In order to avoid any interference in pregnancy upon repeated sampling, the pregnant mice were sampled only once at 1 h post-treatment on day 5. BKI-treatment for 5 days resulted in a stable compound distribution over time with plasma levels between 0.14 and 4.95 μM in non-pregnant mice (Fig. 4). The mean (± standard deviation) plasma concentration of BKI-1708 in the pregnant mice sampled 1 h after the fifth treatment was 1.11 ± 0.75 μM, while the corresponding value in non-pregnant mice was in a similar range (1.42 ± 0.34 μM).

To determine whether BKI-1708 would exert potential embryotoxicity during the first 96 h of embryo development, fertilized zebrafish embryos were exposed to different BKI-1708 concentrations ranging from 0.2 to 50 μM, and zebrafish survival and malformations were evaluated daily by light microscopy in a blinded manner. Concentrations of 2 μM or below did not interfere with embryo development, while higher concentrations had detrimental effects. The score sheets are depicted in Suppl. Table 1, and the scoring summary in Suppl. Table 2.

### 3.4 Efficacy of BKI-1708 in CD1 mice orally infected with TgShSp1 oocysts

**Fig. 5:**
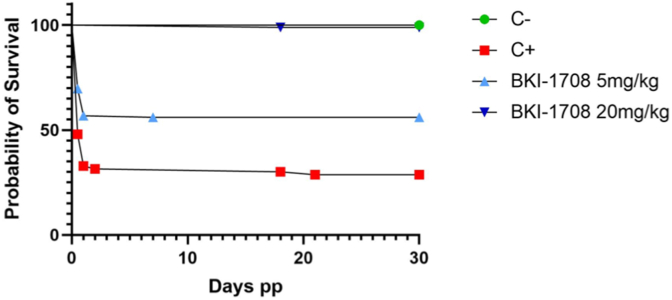
Kaplan-Meier survival curve for toxoplasmosis pregnant mouse model. Survival rates at each time point were plotted in Kaplan-Meier graphs and curves were compared by the Logrank (Mantel-Cox) test. Differences between 20 mg/kg treatment group and positive control curves were highly significant (P < 0.0001).

CD1 mice were orally infected with 100 *T. gondii* TgShSp1 oocysts at day 7 post-mating, and treatment with BKI-1708 (5 or 20 mg/kg/day for 5 days) was initiated 2 days post-infection, while the positive control group was treated with corn oil only. Results are detailed in Table 2 and the Kaplan-Meier survival curves for pups are shown in Fig. 5. Oocyst infection had a profound effect on offspring mice in the positive control group treated with corn oil only, with 68.5 % neonatal mortality (within the first 2 days following birth), and 9.5 % postnatal mortality, amounting up to mortality of 52 out of 73 pups. In comparison, treatment with BKI-1708 at 20 mg/kg/day for 5 days resulted in complete inhibition of neonatal mortality (0%), and only one pup out of 90 died on day 18 p.p. Treatment with 20 mg BKI-1708 also profoundly reduced vertical transmission to 16%, while all pups from the infected and non-treated positive control group were Tg-PCR positive (100% vertical transmission). BKI-1708 dosed at 5 mg/kg seemed not to be effective in preventing vertical transmission, with 130/132 Tg-PCR brain positive pups, however, it had an impact on reducing neonatal mortality (43.1% compared to 68.5% in the positive control) and postnatal mortality (1.3% compared to 9.5% in the positive control group) (Fig. 5).

**Table 2:** Number of mice per group, fertility, postnatal mortality rates and vertical transmission of *T. gondii.*

Group	Treatment	Number of mice	Number of pregnant mice	Fertility rate (%)	Total number of pups/group	Neonatal mortality rate	Postnatal mortality rate	Tg-PCR brain positive adults	Tg-PCR brain positive pups	Vertical transmission rate (%)
Negative control	corn oil	6	4/6	66.6	46	0%	0%	0/6	0/46	0
Positive control	corn oil	12	8/12	66.6	73	68.5%	9.5%	12/12	73/73	100
BKI-1708	5 mg/kg	12	11/12	91.6	132	43.1%	1.3%	11/12	130/132	98
BKI-1708	20 mg/kg	12	7/12	58.3	90	0%	1.1 %	10/12	14/90	16

**Fig. 6:**
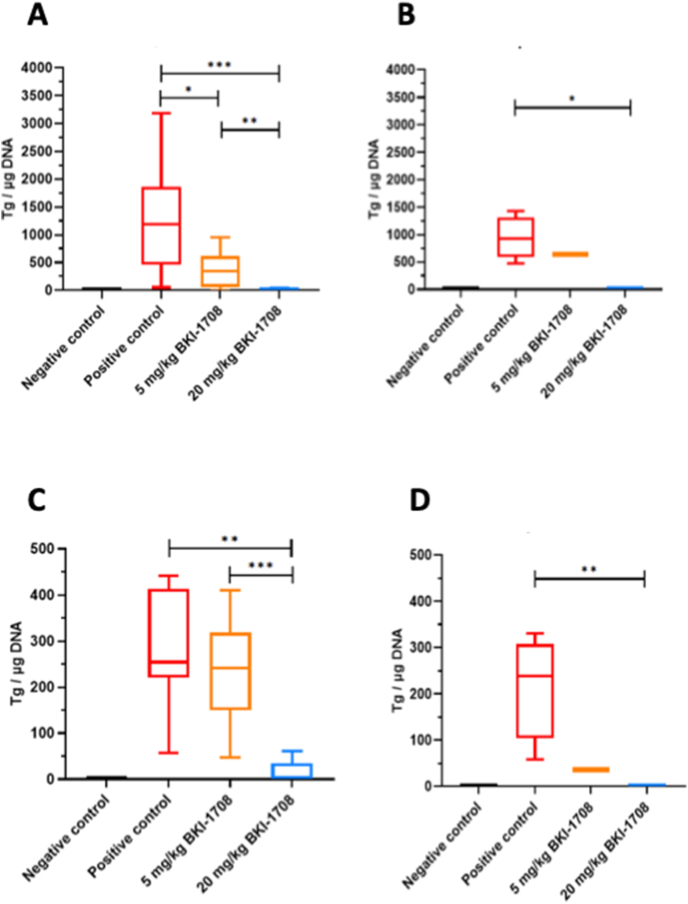
Cerebral (A, B) and ocular cerebral parasite load (C, D) of dams (A, C) and non-pregnant mice (B, D) infected with 100 oocysts of TgShSp1 strain and treated with BKI-1708 at 20 mg/kg/day for 5 days, or with corn oil as placebo (Positive control). Negative control was not infected and treated with corn oil. Eyes and brains were removed after euthanasia and *T. gondii* DNA was extracted and quantified by real-time PCR. Values are shown as box plots. Parasite burden between groups were compared using the Kruskal-Wallis test, followed by the Mann-Whitney-U test. Differences between BKI-1708-treated with 20 mg/kg and non-treated groups were statistically significant in non-pregnant mice and dams. (A) *P = 0.0328; **P = 0.0042, ***P = 0.0003; (B) *P = 0.0018; (C) **P = 0.0042; ***P = 0.0001; (D) **P = 0.005.

Oocyst infection did not induce clinical signs in the adult mice at any time during this experiment. However, virtually all mice from all groups (with few exceptions in the treated groups) tested Tg-PCR positive (Table 2). Clear differences, however, were noted with respect to the cerebral parasite load in both dams and non-pregnant mice, which was highly reduced in the mice treated with 20 mg/kg/day, and less profoundly diminished when BKI-1708 was administered at 5 mg/kg/day (Fig. 6A and B). Similar results were obtained when ocular infection was assessed (Fig. 6C and D).

### 3.5 BKI-1708 is active in BALB/c mice experimentally infected with N. caninum tachyzoites

**Fig. 7:**
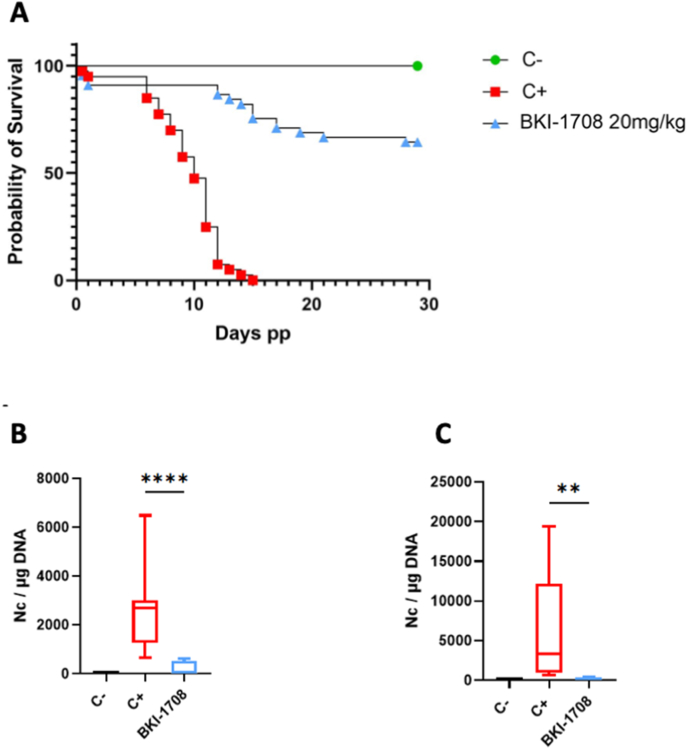
BKI-1708 treatment in the pregnant neosporosis mouse model. BALB/c mice were experimentally infected with 10⁵ NcSpain-7 tachyzoites and treated with BKI-1708 for 5 days at 20 mg/kg/day or with corn oil as placebo (Positive control). Negative control (C-) was not infected and treated with corn oil. A shows Kaplan-Meier survival curves of pups, curves were compared by the Logrank (Mantel-Cox) test. Differences between treated group and C+ curves were highly significant (P < 0.0001). Cerebral parasite burdens in dams (B) and non-pregnant mice (C) were quantified by real time PCR and are shown as box plots. Significant differences in the cerebral parasite burden were observed between BKI-1708-treated mice compared to the C+ group. (B) ****P = 0.0001; (C) **P = 0.0005.

On day 7 post mating, BALB/c mice were infected subcutaneously with 10⁵ *N. caninum* Nc-Sp7 tachyzoites. For this model, the treatment group was dosed only at 20 mg/kg/day for 5 days. Results are summarized in Table 3 and the pup survival curve is shown in Fig. 7A. With one exception, infection with NcSp-7 tachyzoites did not induce clinical signs in the adult mice. One infected pregnant mouse from the non-treated control group stopped gaining weight for 3 consecutive days starting at day 8 of pregnancy and was found presenting dark red vaginal discharge on day 11. The mouse recovered after 2 days, the event was considered an abortion and the mouse was counted as pregnant for the experiment. All other adult mice showed no clinical signs of neosporosis.

**Table 3:** Number of mice per group, fertility, postnatal mortality rates and vertical transmission of *N. caninum.*

Group	Treatment	Number of mice/group	number of pregnant mice	Fertility rate (%)	Total number of pups/group	Neonatal mortality rate	Postnatal mortality rate	Nc**-**PCR positive brain adults	Nc-PCR brain positive **p**ups	Vertical transmission rate (%)
Negative control	corn oil	10	6/10	60	39	0%	0%	0/10	0/43	0
Positive control	corn oil	14	9/14	64.2	41	7.1%	100%	14/14	41/41	100
BKI-1708	20 mg/kg	14	8/14	57.1	46	8.7%	33.4%	4/14	0/46	38

Pups from litters of infected but non-treated mice were underdeveloped, smaller in size and appeared weaker when compared to the uninfected and/or treated groups. As can be seen in Fig. 7A, they all succumbed to infection within 15 days after birth. Treatment with BKI-1708 had no impact on fertility or reproductive parameters, but had a very favorable impact on pup survival, permitting more than 65% survival for BKI-1708 treated pups. Neonatal mortality rate was not impacted by treatment (8.7% in the BKI-group compared to 7.1% in the positive control group treated with corn oil only) but postnatal mortality was reduced from 100% to 33%. Vertical transmission in the BKI-1708 treated group was detected in 38% of pups, while all pups tested positive in the positive control group (Table 3). *N. caninum* DNA was detected in all brains of non-treated control mice, but only in 4 of a total of 14 brains of treated mice. BKI-1708 treatment evidently reduced the cerebral parasitic burden in pregnant and non-pregnant mice as depicted in Fig. 7 B, C.

### 3.6 BKI-1708 treatment alters anti-Toxoplasma and anti-Neospora IgG responses in pregnant and non-pregnant mice

As shown in Table 4, all mice from the non-treated positive control groups of the toxoplasmosis and the neosporosis model were seropositive. In the treatment groups of mice infected with *T. gondii* (n = 12 each), 2 and 1 mouse were seronegative in the 5 and 20 mg/kg/day treatment group, respectively. In the neosporosis model, *Neospora*-specific IgG could not be detected in 2 of the 14 BKI-treated mice (Table 4).

**Table 4:** Number of seropositive mice in the different treatment groups of the toxoplasmosis and neosporosis mouse models.

*In vivo* study	Group	total mice/group	Seropositive for Tg/Nc
Toxoplasmosis (TgShp1)	Negative control	6	0
	Positive control	12	12
	BKI-1708 5 mg/kg	12	10
	BKI-1708 20 mg/kg	12	11
Neosporosis (NcSp-7)	Negative control	10	0
	Positive control	14	14
	BKI-1708 20 mg/kg	14	12

**Fig. 8:**
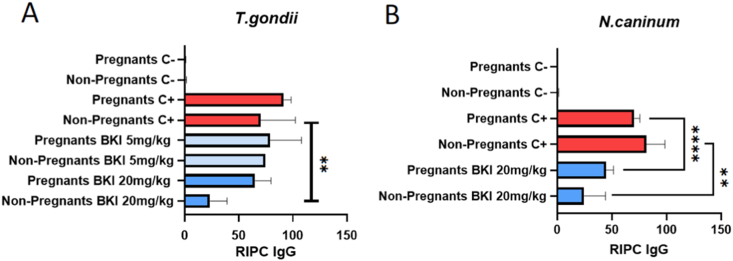
Anti-*T. gondii* and anti-*N. caninum* serum IgG titers quantified by ELISA in mice infected with (A) *T. gondii* or (B) *N. caninum;* RIPC = relative index percentage; (A) **P = 0.0012; (B) **P = 0.0043; ****P = 0.0001.

The quantification of the *Toxoplasma* and *Neospora*-specific IgG responses in dams and non-pregnant mice of the different experimental groups is shown in Fig. 8. Anti-*Toxoplasma* IgG titers in the non-treated positive control groups and the groups treated with BKI-1708 at 5 mg/kg/day were similar, while the BKI-1708 treatment at 20 mg/kg/day resulted in a significantly decreased anti-*Toxoplasma* IgG response in non-pregnant mice, but not in dams. Anti-*Neospora*-IgG responses were significantly lower in BKI-1748 treated non-pregnant mice and dams compared to those from the non-treated positive control groups (Fig. 8).

## 4 Discussion

The present study reports the efficacy of BKI-1708 against *T. gondii* and *N. caninum* infection *in vitro* and in pregnant and non-pregnant mice. *In vitro*, BKI-1708 treatment inhibited proliferation of Tg-β-Gal and Nc-β-Gal tachyzoites with IC_50_ values in the sub-micromolar range. Both pre- and post-infection IC_50_ values for Tg-β-Gal tachyzoites (122 nM and 327 nM) were substantially lower compared to Nc-β-Gal tachyzoites (481 nM and 964 nM), suggesting that *T. gondii* tachyzoites are more susceptible to BKI activity. Such difference in susceptibility in these closely related parasites was also observed for other CDPK1 inhibitors including another 5-aminopyrazole-4-carboxamide compound BKI-1748 (Imhof et al., 2021) and the pyrazolo(2,3-d)pyrimidine compound BKI-1294 (Winzer et al., 2020b).

BKI-1708 was more effective when added concomitantly with infection compared to when added at 3 h post-infection. This could reflect the fact that BKI-1708 is targeting the apicomplexan kinase CDPK1, which is crucially involved in microneme exocytosis, host cell invasion and egress (Ojo et al., 2014; Chan et al., 2023). Thus, parasites treated concomitantly with infection were inhibited in host cell invasion, whereas already intracellular parasites exposed to the drug only after infection were less affected. However, CDPK1 is most likely not the only protein targeted by BKI-1708. Earlier investigations with BKI-1748 indicated that also other putative targets associated with important metabolic pathways could also be affected by BKIs, not only in *N. caninum*, but also in *Cryptosporidium parvum* (Ajiboye et al., 2024). Moreover, related pathways could also be affected in zebrafish embryos and thus contribute to detrimental effects seen at higher drug concentrations (Müller et al., 2022).

In accordance with previous studies employing both 5-aminopyrazole-4-carboxamide as well as pyrazolo(2,3-d)pyrimidine-based BKIs, electron microscopy confirmed that BKI-1708 also caused the formation of intracellular multinucleated complexes (MNCs). This drug-induced stage has been reported not only in *T. gondii* and *N. caninum* (Winzer et al., 2020b; Imhof et al., 2021) but also in other apicomplexans treated with BKIs including *Sarcocystis neurona* (Ojo et al., 2016) and *Besnoitia besnoiti* (Jiménez-Meléndez et al., 2017). TEM demonstrated that BKI-1708 does not notably affect DNA replication and nuclear division, as suggested by the progressive increase of nuclei formed within these MNCs, but inhibited the final steps of cytokinesis involved in the formation of daughter tachyzoites, resulting in an accumulation of newly formed zoites within a large complex. These newly formed zoites, however, exhibited mitochondria with a largely intact mitochondrial matrix, indicating that they were not seriously metabolically impaired. Thus, *in vitro* BKI-1708 exhibits largely parasitostatic activity *in vitro*, and does not have an immediate lethal effect on *T. gondii* tachyzoites. In *N. caninum* exposed to the related pyrazolo(2,3-d)pyrimidine compound BKI-1294, differential proteomics of tachyzoites and MNCs revealed that more than half of the identified proteins exhibited downregulated expression in MNCs as compared to tachyzoites. Only 12 proteins were upregulated, the majority of them containing SAG1 related sequence (SRS) domains, and some also known to be expressed in bradyzoites. Thus, MNCs share some bradyzoite-like features, but may constitute a stage that is formed upon drug pressure (Winzer et al., 2020c). Interestingly, our TEM observations show that *T. gondii* MNCs induced by exposure to BKI-1708 form a cyst-wall like structure surrounding the entire MNC, similar to *T. gondii* tissue cysts formed by bradyzoites. Multinucleated forms of *T. gondii* have been described by Sugi et al. upon treatment with a BKI named 1NM-PP1, which targeted *T. gondii* mitogen-activated protein kinase-like 1 (TgMAPKL-1) (Sugi et al., 2016). In another study, a targeted deletion of the *rab11a* gene in *T. gondii* (TGME49_289680) also induced similar multinucleated parasite structures (Agop-Nersesian et al., 2009). In addition, treatment of *T. gondii* with diclazuril, a triazinone derivative that is effective against intracellular stages of *Eimeria* and *Isospora* spp, was also reported to result in the formation of multinucleated forms similar to the ones observed herein (Lindsay et al., 1995). However, diclazuril is not a kinase inhibitor but acts on enzymes of the respiratory chain and on dihydrofolate reductase (DHFR) (Lindsay et al., 1995). For BKI-1294, MNC formation was shown to be a reversible process, with tachyzoites re-emerging after several days of culture in the absence of drug pressure (Winzer et al., 2020a). It is conceivable that this is also the case for BKI-1708.

Prevention of congenital transmission of toxoplasmosis and neosporosis is crucial since a direct impact of these two parasitic diseases is abortion. Thus, an ideal compound must be safe for use during pregnancy. BKI-1708 had no toxic effects on zebrafish early embryonic development at a concentration of 2 μM and below. Moreover, our pharmacokinetic study in mice treated with BKI-1708 at 20 mg/kg/day for 5 days resulted in plasma levels ranging from 0.14 to 4.95 μM, with a mean plasma concentration of 1.04 ± 0.78 μM. For each time point, plasma levels were below 2 μM, indicating that at that dosage the compound would be safe and not interfere with pregnancy outcome. In addition, the drug is highly plasma-protein bound (>96%), reducing the free drug concentrations that drive toxicity, and was safe for mouse fetal development over the course of BKI-1708 treatment.

To assess *in vivo* efficacy against *T. gondii* infection, outbred CD1 mice were infected with 100 TgShSp1 oocysts, while inbred BALB/c mice experimentally infected with 10⁵ *N. caninum* Sp-7 tachyzoites were used to assess the effects of the drug against neosporosis. These infection doses were previously shown not to cause clinical symptoms in dams but to still induce vertical transmission (Imhof et al., 2021). In the toxoplasmosis model, infection with oocysts leads to a delayed establishment of the disease as inoculated sporozoites must undergo differentiation into tachyzoites, whereas in the neosporosis model 10⁵ tachyzoites were directly inoculated into mice via the subcutaneous route, most likely resulting in a faster progression of the disease and consequently higher cerebral parasite load in shorter time. Thus, direct comparisons should not be drawn between the two models.

BKI-1708 treatment at 5 mg/kg/day for 5 days resulted in a reduced cerebral parasite burden in the dams but did not protect the offspring from vertical transmission. In contrast, treatment at 20 mg/kg/day resulted in profound reduction in the numbers of congenitally infected pups. In addition, the cerebral and ocular parasite loads in treated non-pregnant mice and dams were significantly reduced compared to the control groups. Similar results had been achieved previously with BKI-1748, also at 20 mg/kg/day for 5 days (Imhof et al., 2021). However, in comparison to BKI-1748, BKI-1708 treatment resulted in improved pup survival during the postnatal period (after 2 days post-partum). In previous experiments, treatments of *T. gondii* oocyst infected mice with BKI-1294, which was highly effective against established experimental toxoplasmosis (Doggett et al., 2014), were sufficient to abolish clinical signs in all offspring and resulted in only 4 out of 55 (7%) pups with detectable *T. gondii* DNA in their brain (Müller et al., 2017b). However, BKI-1294 exhibited less favorable pharmacokinetic properties and had been dosed at 50 mg/kg/day for 5 days.

BKI-1708 (20 mg/kg/day for 5 days) was also effective in protecting offspring from *N. caninum* infection and post-partum mortality, increasing pup survival to 62%, while in the control group all mice succumbed to infection by day 15 post-infection. Similar results regarding pup survival were observed with BKI-1748 dosed at 20 mg/kg/day for 5 days (Imhof et al., 2021), but BKI-1748 was less effective in inhibiting vertical transmission of *N. caninum* (14 out of 34 pups were *Neospora* positive) (Imhof et al., 2021). Upon treatment with BKI-1708 all surviving pups were PCR-negative. BKI-1294, assessed in a similar model for experimental neosporosis infection and administered at 50 mg/kg/day for 6 days had also resulted in strongly reduced vertical transmission of *N. caninum* and a pup survival rate of 80% (Winzer et al., 2015). Additionally, studies on BKI-1517 and BKI-1553, both dosed at 20 mg/kg/day for 6 days, showed that (i) BKI-1517 significantly inhibited the vertical transmission of *N. caninum* to pups and increased the rate of survival of offspring, however exhibited clear interference with pregnancy; and (ii) BKI-1553 was less detrimental to fertility and also provided significant, but clearly less pronounced, protection of dams and offspring (Müller et al., 2017a). The numbers of offspring in groups treated with BKI-1708 for both models were virtually identical to those in untreated controls, confirming that treatment bears no impact in fertility or number of pups, not interfering with pregnancy outcome.

## 5 Conclusions

For both models, BKI-1708 treatment resulted in a dramatic reduction of the cerebral parasite load, and significantly prevented vertical transmission of *T. gondii* and *N. caninum,* reducing the numbers of congenitally infected pups by 84 and 62% compared to the controls, respectively. Cerebral parasite loads, and ocular infections in the case of toxoplasmosis, were also significantly reduced. BKI-1708 administered at 20 mg/kg/day for 5 days, established an average threshold below 2 μM, rendering this compound highly active while not inducing embryotoxicity, thus safe to be applied during pregnancy. In vitro, exposure of *T. gondii* resulted in the formation of multinucleated complexes, as previously described for *N. caninum*. However, in the case of *T. gondii* ME49, exposure to BKI-1708 *in vitro* resulted in the formation of a cyst wall like structure.

## Funding

This study was financed by the 10.13039/100000001Swiss National Science Foundation (10.13039/501100001711SNSF) grant 310030_214897, the 10.13039/100000002National Institutes of Health (10.13039/100000002NIH) grants R01AI089441, R01AI111341, R01HD080670, R01AI155412, R01HD102487, R21AI123690, and R21AI140881, and 10.13039/100000199United States Department of Agriculture, 10.13039/100005825National Institute of Food and Agriculture grants # 2019-07512 and # 2014–06183. Maria Ferreira was funded by a Swiss Government Excellence Fellowship, and Kai Haenggeli was supported by the Uniscientia Foundation.

## CRediT authorship contribution statement

**Maria Cristina Ferreira de Sousa:** Writing – review & editing, Writing – original draft, Methodology, Investigation, Formal analysis, Data curation. **Dennis Imhof:** Writing – review & editing, Supervision, Methodology, Investigation, Formal analysis, Data curation, Conceptualization. **Kai Pascal Alexander Hänggeli:** Writing – review & editing, Methodology, Investigation. **Ryan Choi:** Writing – review & editing, Methodology, Formal analysis. **Matthew A. Hulverson:** Writing – review & editing, Methodology, Investigation. **Samuel L.M. Arnold:** Writing – review & editing, Methodology, Investigation. **Wesley C. Van Voorhis:** Writing – review & editing, Validation, Funding acquisition, Conceptualization. **Erkang Fan:** Writing – review & editing, Resources, Methodology, Investigation. **Sánchez-Sánchez Roberto:** Resources, Methodology, Investigation. **Luis M. Ortega-Mora:** Writing – review & editing, Resources, Methodology, Funding acquisition, Data curation. **Andrew Hemphill:** Writing – review & editing, Visualization, Resources, Methodology, Funding acquisition, Conceptualization.

## Declaration of competing interest

none, no conflict of interest.
