# Supplementary material for: Efficacy of the bumped kinase inhibitor BKI-1708 against the cyst-forming apicomplexan parasites Toxoplasma gondii and Neospora caninum in vitro and in experimentally infected mice
Source: Int J Parasitol Drugs Drug Resist. 2024 Jun 19;25:100553. doi: 10.1016/j.ijpddr.2024.100553 (PMC11254172; doi:10.1016/j.ijpddr.2024.100553)
Supplement: Multimedia component 1 [file mmc1.docx]

**Supplementary Table 1**. Score sheet for treated Zebrafish embryos with BKI-1708 concentrations ranging from 0.2 μM to 50 μM. A total of 20 eggs were microscopically assessed for each concentration or the respective negative or solvent controls. Controls are depicted in A-C and treated embryos in D-K. Each table represents the treatment wells in a 24-well-plate, where x refers to dead embryos; h are hatched eggs; p e stands for pericardial edema; coag for coagulated embryo (dead); m and tm refer to malformation and tail malformation, respectively.


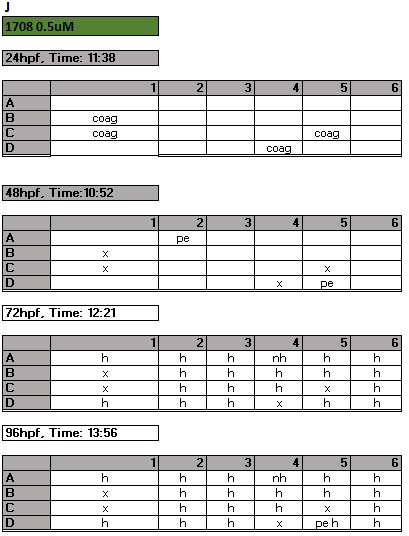

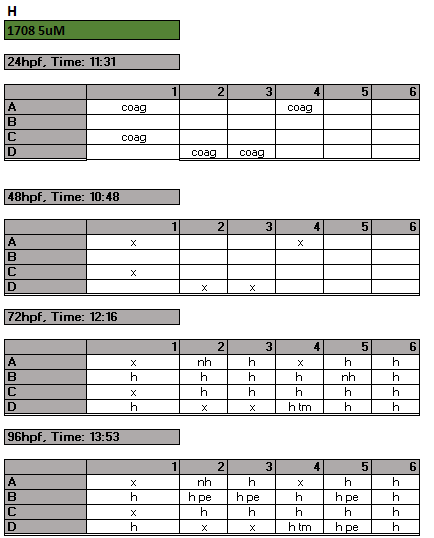

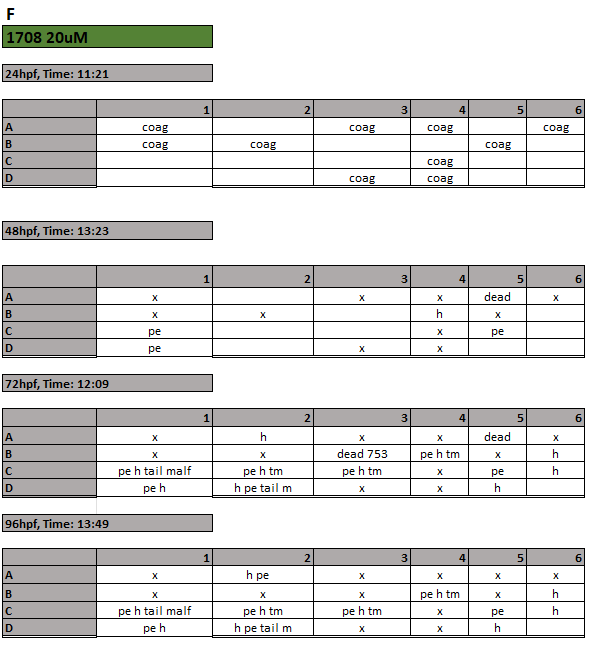

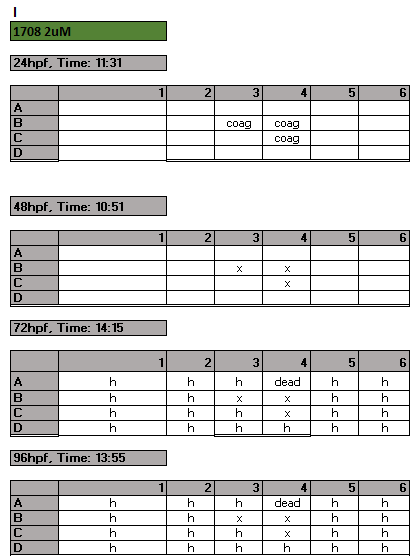

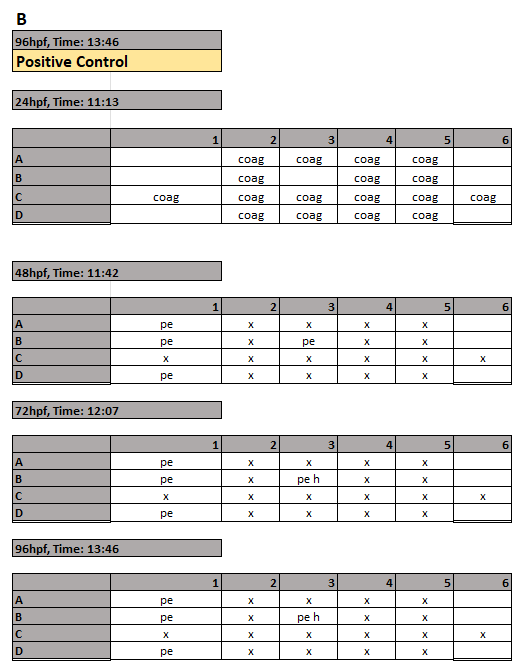

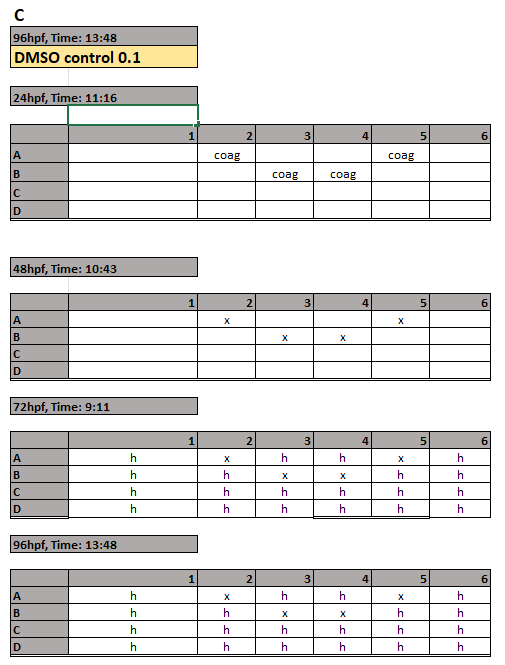

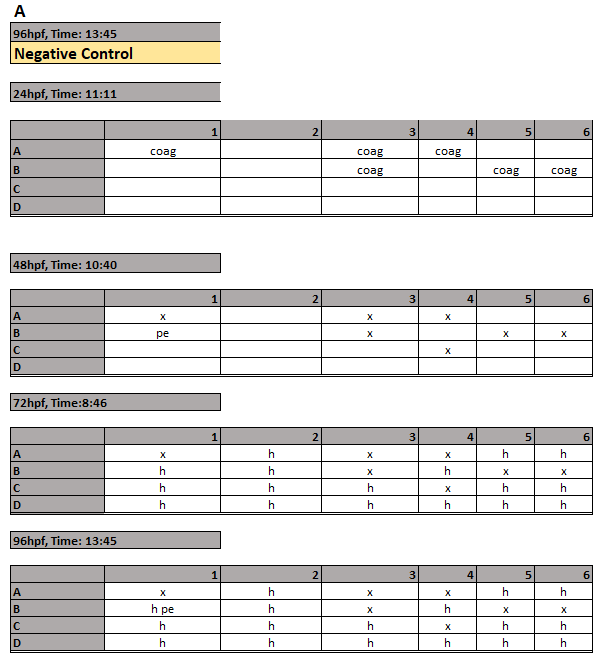


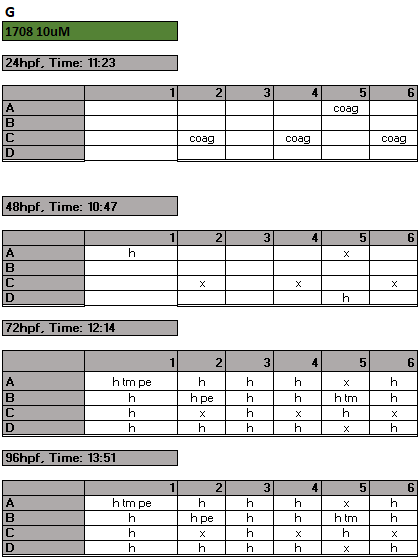

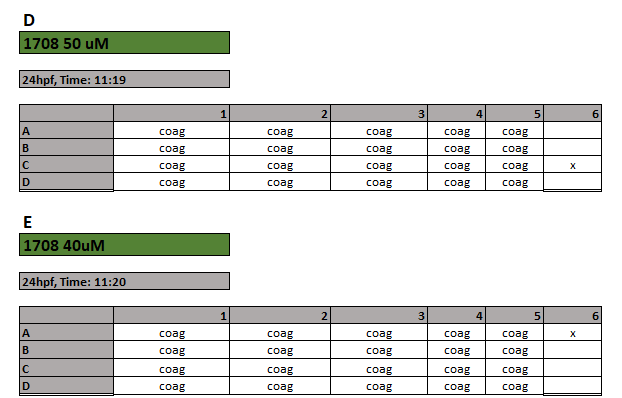


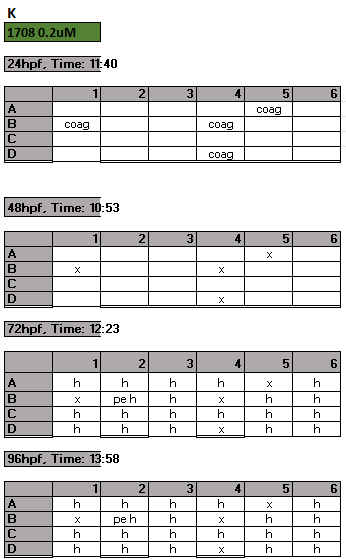


**
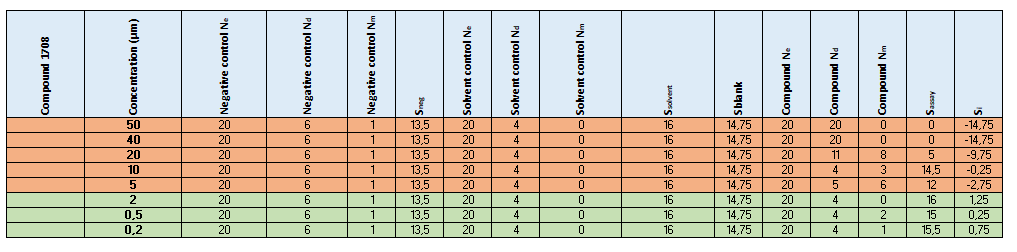
Supplementary Table 2.** Scoring summary table for BKI-1708 treated zebrafish embryos. In each assay, a score of −1 was assigned for death during the 96hpf, each malformation seen on a hatched embryo received a score of −0.5, including non-hatched embryos at 96hpf. Thus, the score for a given assay (Sassay) was calculated by subtracting the scores for dead embryos (Nd) and the scores for malformations (Nm divided by half) from Ne. The mean control score (Smean) was determined by calculating the mean of the negative control score (Sneg) and the solvent control score (SDMSO). The overall impact score for a given drug concentration (Si) was finally calculated by subtracting Smean from the Sassay achieved with the test dilution. A negative Si indicates interference, and a Si of 0 or higher implies no interference in early embryo development. Negative control Ne: number of eggs introduced at the beginning of the experiment; Negative control Nd: number of deaths of embryos accounted at 96hpf; Solvent control Nm: number of malformations accounted at 96hpf (the embryo must be alive, malformations proceeded by death until the 96hpf are considered death not malformations.
